# Closing the quality gap: analysis of implementation barriers and interventions for postoperative exercise rehabilitation for patients with radiofrequency ablation for atrial fibrillation

**DOI:** 10.3389/fcvm.2026.1757839

**Published:** 2026-06-16

**Authors:** Bingqing Lu, Yajun Sun, Lei He, Sijia Sun, Jiajia Wu, Bo Chen, Hongyou Fan, Zhi Zhao

**Affiliations:** 1 Department of Cardiology, The First Affiliated Hospital of Soochow University, Suzhou, China; 2 Department of Cardiovascular Surgery, The First Affiliated Hospital of Soochow University, Suzhou, China; 3 Department of Infectious Diseases, The First Affiliated Hospital of Soochow University, Suzhou, China

**Keywords:** atrial fibrillation, exercise-based cardiac rehabilitation, obstacle analysis, radiofrequency ablation, review criteria

## Abstract

**Background:**

Atrial fibrillation patients often experience issues such as recurrence and reduced exercise tolerance following radiofrequency catheter ablation, which adversely affect postoperative recovery and long-term prognosis. Although current guidelines recommend exercise-based rehabilitation for these patients, its implementation in clinical practice remains suboptimal.

**Objective:**

This study aims to summarize the best evidence regarding post-ablation exercise rehabilitation for atrial fibrillation, establish structured quality evaluation metrics, and identify barriers in clinical practice, thereby informing standardized management of exercise rehabilitation.

**Methods:**

This study included evidence synthesis, baseline audit, barrier analysis, and strategy development. Guided by the Joanna Briggs Institute (JBI) evidence-based healthcare model, formulated a clear evidence question and established an evidence-based team responsible for literature retrieval and quality assessment, established quality indicators and review methods, analyzed the barriers and facilitators factors, and formed evidence-based practice strategies.

**Results:**

A total of sixteen evidence points across four areas, including exercise timing, assessment, prescription, monitoring, and follow-up. Twenty quality indicators were subsequently developed. Clinical quality evaluation revealed several implementation barriers, such as insufficient localization, inadequate knowledge and skills among healthcare personnel, patient misconceptions, and an incomplete organizational support system. These represent multi-level obstacles.

**Conclusion:**

A significant evidence-practice gap exists in post-ablation exercise rehabilitation. The effective translation of evidence into practice entails a thorough analysis of implementation barriers and the subsequent formulation of tailored, evidence-based strategies to facilitate this process.

## 1 Introduction

Atrial fibrillation is the most common supraventricular arrhythmia (1) and is clinically characterized by symptoms such as palpitations, dizziness, and fatigue (2). It elevates the risk of mortality (3), heart failure (4), and stroke (5). The global prevalence of atrial fibrillation continues to rise, imposing a substantial clinical and economic burden worldwide (6, 7). Radiofrequency ablation is a first-line treatment for atrial fibrillation that effectively reduces symptom burden and maintains sinus rhythm (8, 9). Nevertheless, postoperative recurrence remains a significant clinical issue, with the CIRCA-DOSE study confirming early recurrence in 61% of patients (10).

A key modifiable factor is physical inactivity. Existing evidence indicates that a sedentary lifestyle is an independent risk factor for atrial fibrillation recurrence (11–13). Furthermore, studies indicate that reduced left atrial compliance following ablation leads to decreased cardiac output, which in turn lowers patients' activity levels (14, 15). This creates a vicious cycle: the very procedure intended to treat atrial fibrillation temporarily impairs cardiac function, leading to reduced activity, which then promotes recurrence. Therefore, breaking this cycle through regular exercise training is crucial for mitigating the risk of postoperative recurrence and ensuring recovery.

Exercise-based Cardiac Rehabilitation (ExCR) has become a key component of postoperative care for atrial fibrillation patients, with evidence indicating its role in reducing recurrence risk after ablation (16). Engaging in moderate-to-high intensity exercise training with a weekly volume of 1,000–1,500 METs has been shown to reduce atrial fibrillation risk by 10% (17). This protective effect is attributed to improved endothelial function, alleviated myocardial ischemia, and suppression of atrial electrical remodeling (18). Furthermore, ExCR enhances physical condition, exercise tolerance, and cardiac function in post-ablation patients by improving cardiac autonomic regulation and skeletal muscle oxygen utilization, which collectively improve cardiopulmonary endurance (19, 20).

Despite established guideline recommendations supporting the use of regular ExCR to improve clinical outcomes in atrial fibrillation patients (1), this evidence-based strategy has not been consistently integrated into routine care. The challenge lies not in a lack of theoretical support, but in the complex process of translating standardized recommendations into effective practice for a diverse patient population. On one hand, patients frequently struggle to adhere to generic exercise prescriptions due to significant individual differences in exercise tolerance and cardiac function (21), a reality reflected in the notoriously low long-term adherence rates, fewer than 5% of cardiovascular patients sustaining their regimens (22). On the other hand, and more fundamentally, these patient-level challenges are exacerbated by a common deficit in structured clinical support systems. The frequent absence of integrated protocols for initial functional assessment, personalized dose adjustment, and proactive long-term follow-up within routine care settings means that patients are often left to navigate their rehabilitation without the necessary professional guidance to overcome individual barriers. Therefore, addressing the persistent gaps in evidence-based post-ablation exercise rehabilitation requires implementing individualized interventions to promote effective translation into practice.

This study was guided by the JBI Model of Evidence-Based Healthcare (23). It established an evidence-based team, identified a clinical question, conducted a systematic literature search, appraised evidence quality, and synthesized the best available evidence. Subsequently, quality indicators were developed, review methods were clarified, and a clinical audit was conducted. This study was also guided by the integrated-Promoting Action on Research Implementation in Health Services (i-PARIHS) framework (24). Barriers and facilitators were analyzed across three dimensions, namely innovation, recipients, and context, and corresponding implementation strategies were developed. Continuous evaluation and dynamic adjustment of these strategies aim to support evidence-informed practice for exercise rehabilitation in patients after atrial fibrillation ablation.

## 2 Material and methods

This study included three main steps: (a) evidence synthesis and quality indicator development; (b) baseline audit and barrier analysis; (c) development of implementation strategies based on the i-PARIHS framework.

### 2.1 Identify problems

The evidence-based question was structured using the PIPOST model (25). Population (P): Patients with atrial fibrillation after radiofrequency ablation. Intervention (I): exercise rehabilitation. Professional (P): Nurses, doctors, and rehabilitation therapists. Outcome (O): Recurrence rate, cardiopulmonary endurance, atrial fibrillation burden, resting heart rate, and exercise heart rate. Setting (S): Family, community, hospital, and cardiac rehabilitation center. Type of evidence (T): clinical guidelines, systematic reviews, expert consensus, evidence summary, and meta-analysis.

### 2.2 Build a team

#### 2.2.1 Establish an evidence-based team

This study established a multidisciplinary evidence-based nursing team comprising eight members to facilitate the clinical translation and implementation of evidence on post-ablation exercise rehabilitation in atrial fibrillation. The team included two head nurses trained in evidence translation methodology through workshops organized by the Fudan University Center for Evidence-Based Nursing, who oversaw project coordination and provided methodological guidance. Also participating were one cardiologist responsible for study design and feasibility evaluation, one sports rehabilitation specialist who contributed to program design and exercise supervision, one cardiovascular nursing expert leading the clinical integration of evidence, and three clinical nurses from the cardiology department implementing the project and collecting data.

### 2.3 Retrieve literature

**Figure 1:**
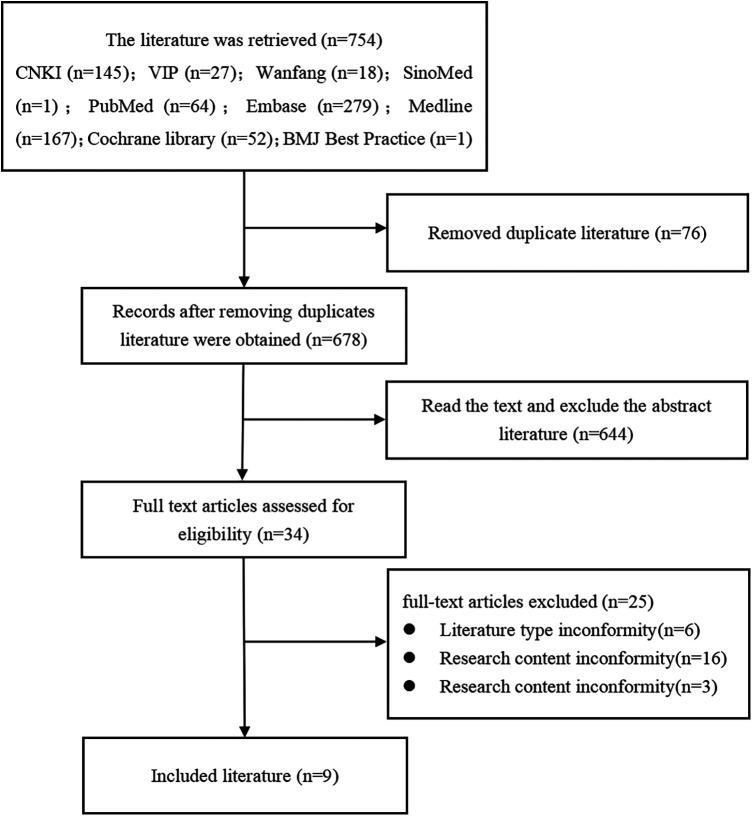
flow chart of literature screening.

A comprehensive search was conducted following the “6S” evidence pyramid model to identify evidence on home-based exercise rehabilitation after radiofrequency ablation for atrial fibrillation (26). Following the 6S evidence pyramid, we prioritized high-level evidence based on the PICO question, and only retrieved primary studies when higher-level evidence was insufficient. Sources included Up To Date, BMJ Best Practice, ACSM, AHA, ACC, ESC, as well as major Chinese and international databases such as PubMed, Embase, Medline, Cochrane Library, Chinese Medical Association (CMA), China National Knowledge Infrastructure (CNKI), Sinomed, VIP, and Wanfang Data. Search terms encompassed: Atrial Fibrillation, Arrhythmias, Radiofrequency Ablation, Catheter Ablation, Ablation Techniques, Exercise-based Cardiac Rehabilitation, Exercise, Activity, Management, Recurrence, Cardiorespiratory Fitness, Symptom, Burden, Heart rate, Guidelines, Systematic Reviews, Evidence Summaries, Expert Consensus, and Meta-Analysis. The search covered records from database inception until June 1, 2025. From 754 initially identified articles, 9 met the inclusion criteria after removing duplicates and irrelevant studies, comprising 5 guidelines, 3 expert consensuses, and 1 systematic review (Figure 1).

Inclusion Criteria: Patients diagnosed with atrial fibrillation based on the 2023 ACC/AHA/ACCP/HRS guideline (27); History of radiofrequency catheter ablation for atrial fibrillation; Study focused on exercise-based cardiac rehabilitation in atrial fibrillation; Publication types including clinical guidelines, evidence summaries, systematic reviews, expert consensuses, meta-analyzes, and clinical decision support tools. Exclusion Criteria: Full text not accessible; Secondary publications such as guideline interpretations or consensus commentaries.

### 2.4 Evaluate and summarize the best evidence

Guidelines were appraised using the Appraisal of Guidelines for Research and Evaluation II (AGREE II) instrument (28), while expert consensus and systematic reviews were evaluated according to the Joanna Briggs Institute (JBI) critical appraisal tools (2016) (29). Four researchers trained in evidence-based methods at Fudan University independently assessed the guidelines. The remaining literature was evaluated by two researchers, with any disagreements resolved through discussion with a third reviewer. Evidence was summarized and graded by two independent reviewers using the JBI evidence pre-leveling system (2014) (30), which categorizes evidence into Levels 1–5. Discrepancies were resolved by consensus or consultation with a third reviewer.

### 2.5 Establishment of evidence-based review indicators and baseline review

Members of the Evidence-Based Practice Group, in collaboration with nine expert panel members, convened an expert panel meeting. Drawing upon clinical scenarios and professional judgment, they developed corresponding review criteria, subjects, tools, and methods for each of the 16 previously compiled evidence items based on the FAME principles (23). Finally, members of the Evidence-Based Practice Group conducted the clinical baseline review.

This baseline review included all eligible patients and healthcare providers in the cardiovascular medicine department of a tertiary hospital in a Chinese city from January to June, 2025. A total of 30 patients and 17 professional healthcare workers who met the inclusion and exclusion criteria were enrolled. No sample size calculation was performed as this was a descriptive audit of current practice, not a hypothesis-testing study. This research was approved by the Ethics Committee of the First Affiliated Hospital of Soochow University (Approval No.2025640).

Inclusion Criteria for patients: diagnosed with atrial fibrillation according to the 2023 ACC/AHA/ACCP/HRS guideline (27); at least one month post radiofrequency ablation; aged 18 years or older, mentally clear, without cognitive impairment or organic mental disorders; and who provided informed consent. Exclusion Criteria for patients: severe organic diseases or major cardiopulmonary dysfunction such as heart failure, malignant arrhythmias, or primary tumors; conditions impairing exercise capacity, including orthopedic or thrombotic diseases; and concurrent participation in other clinical trials.

Inclusion criteria for clinical nurses: Hold a Nurse Practice certificate of the People's Republic of China; Have been engaged in clinical nursing work for cardiovascular diseases for at least one year; Informed consent and voluntary participation in this study. Exclusion criteria for clinical nurses: Clinical training nurses and standardized training nurses; Nurses who were not on duty during the investigation period due to illness, personal leave, or going out for further studies.

### 2.6 Identification of implementation barriers

Following the baseline review, an analysis of enabling and barrier factors was conducted based on the i-PARIHS model. The core perspective of the i-PARIHS framework posits that the success of evidence application hinges on four key elements: Enablers, innovation, Recipients, and Context. Among these, “Enablers” is not a standalone dimension but rather an active element permeating the entire evidence application process. It facilitates effective collaboration among individuals, groups, and teams to achieve shared objectives. This study employed the Facilitation Checklist within the i-PARIHS framework as its research tool. Using brainstorming techniques, it systematically analyzed facilitators and barriers for indicators with compliance rates below 60%. Explanations were provided for the facilitators and barriers associated with each item, and action strategies were formulated.

Guided by the i-PARIHS framework, this study conducted a multidimensional analysis of barriers and facilitators affecting the adoption of audit criteria with compliance rates below 60%, focusing on the innovation, recipients, and context. The findings provide a structured pathway to support the clinical implementation of exercise rehabilitation in post-ablation atrial fibrillation care.

## 3 Results

### 3.1 Included evidence

This study summarized evidence on exercise rehabilitation for atrial fibrillation patients after radiofrequency ablation, identifying 16 key recommendations across four domains: exercise timing and assessment, prescription, monitoring, and follow-up, as presented in Table 1.

**Table 1:** Evidence summary of best practices for ExCR in patients post-atrial fibrillation catheter ablation.

Category	Content of evidence	Level	Recommendation level
ExCR assessment and selection of timing	1. ExCR requires pre-participation assessment to exclude absolute contraindications (13, 14).	1	A
	2. Physical activity levels were assessed pre-exercise using the International Physical Activity Questionnaire (IPAQ). For eligible patients without contraindications, evaluations including cardiopulmonary exercise test (CPET) or 6-minute walk test (6MWT) can also be conducted (14).	1	A
	3. Patients without recurrence within 1 month post-ablation may gradually resume physical activity (13).	5	C
	4. Exercise should be delayed until at least two elimination half-lives after administration of propafenone or other class I antiarrhythmic drugs (13).	5	C
ExCR prescription	5. Personalized exercise prescriptions, incorporating diverse modalities, are recommended, with intensity tailored based on individual patient characteristics and assessment findings (14).	5	C
	6. Moderate-to-vigorous intensity exercise training is recommended weekly (15, 16), with aerobic intensity assessed using the Borg Rating of Perceived Exertion (RPE) scale or the talk test (14).	1	A
	7. The recommended exercise regimen comprises a combination of aerobic and resistance training. Frequency: At least 3–5 sessions per week (13, 17). Duration: ≥ 30 min per session (13, 17), accumulating to ≥ 210 min weekly (8, 18). Aerobic Training (13, 14): Includes activities such as walking, running, swimming, and cycling. It is advisable to initiate aerobic exercise using short-bout interval training and progressively transition to sustained continuous exercise. Resistance Training (13, 14): Includes exercises targeting major muscle groups (e.g., modified pull-ups, crunches, push-ups). Perform 1–3 sets of 8–15 repetitions per exercise, with training for the same muscle group occurring no more frequently than every other day (≥ 48 h rest interval).	1	A
	8. For patients with obesity (BMI ≥ 30 kg/m2), a progressive exercise regimen is recommended (19): Initial prescription: Low-intensity exercise for 20 min per session, 3 times per week. Progression target: At least 200 min of moderate-intensity exercise weekly. Weight management goal: Achieve a BMI ≤ 25 kg/m2.	1	B
	9. For older patients (≥ 65 years), moderate-to-high intensity functional balance and strength training (e.g., Tai Chi, Baduanjin or dumbbell exercises) is recommended at least three times per week (13).	1	A
	10. For patients with an elevated resting heart rate (> 80 bpm), mind-body exercises emphasizing breath regulation, such as Baduanjin, Yoga, Standing Meditation and Mindful Walking, are recommended as moderate aerobic activities (14).	5	C
	11. Patients receiving anticoagulant therapy should avoid contact sports or activities with a high risk of trauma (13).	3	A
ExCR monitoring	12. Heart rate should be maintained below 110 bpm during exercise (20).	2	B
	13. During exercise, blood pressure should be maintained at or below 200/100 mmHg in patients with hypertension (17, 18).	5	C
	14. If atrial fibrillation recurs during exercise and is accompanied by symptoms potentially attributable to rapid ventricular rate (e.g., dizziness, palpitations, syncope or fatigue), exercise must be terminated immediately. Following symptom resolution, the patient's eligibility for continuation of exercise rehabilitation should be formally reassessed (14).	5	C
ExCR Follow-up	15. AF management team should guide patients through one-on-one consultations during exercise rehabilitation to master the following postoperative self-management skills: Regular anticoagulation therapy, Recognizing signs of atrial fibrillation recurrence, Safe monitoring during exercise (14).	5	C
	16. Follow-up: Develop discharge plans and follow-up protocols tailored to the patient's condition. Depending on circumstances, follow-up may be conducted through outpatient visits, telephone consultations, or other appropriate methods (14).	5	C

Absolute contraindications (31): Acute cardiac events (e.g., myocardial infarction, unstable angina) within 2 days; new-onset resting ECG ischemic changes; uncontrolled arrhythmias causing symptoms or hemodynamic compromise; decompensated heart failure, active endocarditis or myocardial/pericardial inflammation; concurrent acute systemic diseases (e.g., infection, renal failure, hyperthyroidism) and acute pulmonary embolism; resting heart rate > 120 beats/min, severe aortic stenosis, and patient non-compliance.

### 3.2 Quality indicators and review findings

The evidence review panel conducted an assessment of evidence applicability based on the FAME principles (32, 33), ultimately excluding 2 pieces of evidence and modifying 1 piece, resulting in the retention of 14 pieces of evidence.

Two evidence items were excluded due to local infeasibility. The propafenone-related timing recommendation was excluded because class III antiarrhythmics are more commonly prescribed in our center. The ambulatory blood pressure monitoring recommendation was excluded because current technology cannot accurately measure BP during dynamic exercise.

One evidence item was modified: the exercise heart rate threshold was raised from < 110 bpm to < 140 bpm (34, 35). This modification is a context-specific, feasibility-driven adaptation based on the FAME assessment. The original evidence for < 110 bpm during exercise derives from strict rate control targets. The < 140 bpm threshold is extrapolated from the HOT CAFE study, which used a target of ≤ 140 bpm during moderate exercise in patients with persistent atrial fibrillation receiving rate control therapy; this study is cited in the 2023 ACC/AHA/ACCP/HRS guidelines (27). However, direct evidence specific to the post-ablation exercise setting for this threshold remains limited. Therefore, this adaptation is based on clinical feasibility, expert consensus, and the principle of lenient, symptom-guided rate control endorsed by the guidelines. This is not a claim of evidence superiority over existing guidelines.

Following the SMART principles, 20 review indicators were developed from 14 evidence items (Table 2). The baseline review revealed that 1 of the 20 indicators achieved a 100% implementation rate, 4 indicators had implementation rates below 60%, and 15 indicators had 0% implementation rates (Table 3).

**Table 2:** Evidence-Based nursing quality indicators and evaluation methods for ExCR after radiofrequency catheter ablation of atrial fibrillation.

Quality indicators	Review object	Review method
1. Attending Physician should conduct pre-exercise screening to identify absolute contraindications.	Attending Physician	Case Query Method
2. Before initiating exercise, nurses assess patients' functional capacity using validated tools such as IPAQ, CPET, or 6MWT.	Nurse	Review the sports assessment form
3. Patients who remain free of atrial fibrillation recurrence at 1 month post-procedure may be considered for initiation of supervised exercise rehabilitation.	Attending Physician	Direct evaluation
4. Healthcare providers develop individualized exercise prescriptions incorporating diverse exercise modalities.	Healthcare providers	Review the exercise prescription assessment form
5. Exercise prescriptions specify modality selection, with intensity individualized through ongoing assessment of patient-specific parameters and functional responses.	Rehabilitation Therapist	Direct evaluation
6.1 Patients engage in prescribed moderate-to-vigorous intensity exercise training weekly.	Patient	Review wearable monitoring devices records or ExCR diaries
6.2 Patients use aerobic exercise intensity using either the Borg RPE scale or the Talk Test.	Patient	Direct evaluation
7.1 Patients perform combined aerobic and resistance training, accumulating ≥ 210 min of moderate-intensity exercise weekly.	Patient	Review wearable monitoring devices records and ExCR diaries
7.2 Aerobic exercise is performed 3–5 days weekly, with each session lasting ≥ 30 min at moderate intensity.	Patient	Review wearable monitoring devices records and ExCR diaries
7.3 Resistance exercises comprise 8 to 15 repetitions per set, with each muscle group trained every other day.	Patient	Review wearable monitoring devices records and ExCR diaries
8.1 Obese patients with body mass index ≥ 30 kg/m^2^ commence a structured exercise progression from thrice-weekly 20-minute low-intensity sessions, gradually advancing to achieve a minimum of 200 weekly minutes of moderate-intensity activity.	Patient	Review wearable monitoring devices records and ExCR diaries
8.2 Obese patients (defined as having a baseline BMI ≥ 30 kg/m²) achieved a BMI ≤ 25 kg/m² following a 3-month exercise intervention.	Patient	Review wearable monitoring devices records and ExCR diaries
9 Older patients aged ≥ 65 years engaged in moderate-to-high intensity functional balance and strength training, such as Tai Chi, Baduanjin, or dumbbell exercises, at least three times per week.	Patient	Review wearable monitoring devices records and ExCR diaries
10 Patients with a resting ventricular rate exceeding 80 bpm were prescribed breath-focused aerobic exercises, such as Baduanjin, Yoga, Standing Meditation, or Mindful Walking.	Patient	Review wearable monitoring devices records and ExCR diaries
11 Patients receiving anticoagulant therapy were advised to avoid contact sports and activities prone to trauma.	Patient	Direct evaluation
12 Patients' heart rates were maintained below 140 bpm during exercise.	Patient	Direct evaluation
13. Patients were aware of the warning symptoms necessitating exercise cessation.	Patient	Direct evaluation
14. Following exercise cessation due to symptoms, the Attending Physician and Rehabilitation Therapist conducted clinical assessments to determine the safety of resuming exercise rehabilitation.	Attending Physician and Rehabilitation Therapist	Review the sports assessment form Direct evaluation
15. The atrial fibrillation management team provided one-on-one counseling during exercise training sessions.	Healthcare providers	View on-site
16. Nurses developed individualized discharge plans and follow-up protocols based on clinical assessments, implementing either in-person clinic visits or telephone consultations according to patient-specific needs.	Nurse	Review post-discharge surveillance registry

**Table 3:** Summary table of compliance with clinical review indicators.

Quality indicators	Number of reviews	Review result			Compliance rate (%)
		Y	N	NA	
Indicator 1	17	0	17	0	0.0%
Indicator 2	17	0	17	0	0.0%
Indicator 3	30	6	24	0	20.0%
Indicator 4	17	0	17	0	0.0%
Indicator 5	17	0	17	0	0.0%
Indicator 6.1	30	2	28	0	7.0%
Indicator 6.2	30	0	30	0	0.0%
Indicator 7.1	30	0	30	0	0.0%
Indicator 7.2	30	0	30	0	0.0%
Indicator 7.3	30	0	30	0	0.0%
Indicator 8.1	2	0	2	28	0.0%
Indicator 8.2	2	0	2	28	0.0%
Indicator 9	12	0	12	18	0.0%
Indicator 10	4	0	4	26	0.0%
Indicator 11	30	30	0	0	100.0%
Indicator 12	30	4	26	0	13.0%
Indicator 13	30	11	19	0	37.0%
Indicator 14	17	0	17	0	0.0%
Indicator 15	17	0	17	0	0.0%
Indicator 16	17	0	17	0	0.0%

Clinical compliance rate = Y (number of reviews)/[Y (number of reviews) + N (number of reviews)].

*Y*, Compliance with quality indicators; *N*, Non-compliance with quality indicators; *NA*, Not applicable.

### 3.3 Obstacle and promoting factor analysis and action strategies

For the quality indicator with an implementation rate of less than 60%, the evidence-based team systematically analyzed the obstructive factors and promoting factors, and designed targeted improvement strategies based on the i-PARIHS framework. This study identified challenges across the innovation, recipient, and context domains during evidence-based practice for post-ablation exercise rehabilitation. At the innovation level, the project itself faces multiple challenges: international experiences prove difficult to effectively adapt to the local context, compounded by a lack of standardized processes and evaluation tools. On the recipient side, healthcare providers exhibit limited willingness to adopt the project due to insufficient knowledge reserves, heavy workloads, and safety concerns. Patient uptake rates remained persistently low, influenced by traditional rehabilitation beliefs, poor treatment adherence, and transportation-related cultural factors. Implementation barriers included inexperience, absence of cross-departmental coordination mechanisms, and difficulties in managing multiple wards (Table 4).

**Table 4:** Obstacle factors, facilitators, and action strategies for ExCR in patients after atrial fibrillation radiofrequency ablation.

Theoretical Framework (i-PARIHS)	Obstacle factors	Promoting factors	Action strategies
Innovation	Evidence primarily originates from international studies, differing from China's clinical practice environment Standardized home exercise rehabilitation protocols for post-atrial fibrillation radiofrequency ablation patients remain unestablished Departments lack unified tools for exercise risk screening and assessment Hospital electronic medical record systems lack structured exercise rehabilitation assessment entries	Evidence largely derives from clinical guidelines and expert consensus with high-grade quality Existing universal assessment tools, such as the 6MWT and Borg Scale The hospital information system platform has reserved interfaces for rehabilitation assessment modules	Conduct a pilot study in 2 wards Promote the integration of structured exercise rehabilitation assessment items into the hospital information system Develop standardized home-based exercise rehabilitation assessment and implementation protocols based on best evidence
Recipients (Healthcare Providers)	Healthcare professionals lack sufficient knowledge and skills in evidence-based practice Heavy clinical workloads limit their capacity and willingness to invest in change Some physicians harbor doubts about the safety and efficacy of home-based exercise Patients transitioning from passive treatment recipients to active decision-makers increases communication and guidance burdens for healthcare providers	All healthcare professionals hold bachelor's degrees or higher and have received systematic training, possessing foundational implementation capabilities Healthcare providers widely recognize the critical role of exercise rehabilitation in postoperative management and demonstrate strong subjective willingness to implement it All key stakeholders involved in the project participated in the preliminary design and discussion process	Survey healthcare providers' specific needs regarding training content and supportive tools Conduct evidence-based practice and specialized exercise rehabilitation skill workshops, and send key personnel for external training Organize departmental group learning sessions to systematically interpret evidence highlights, enhancing understanding and acceptance
Recipients (Patients)	Patients commonly hold the traditional belief that “postoperative rest is necessary,” harboring misconceptions and safety concerns about exercise rehabilitation Lack the ability and information to safely and effectively execute exercise rehabilitation plans in unsupervised settings Some patients reside in remote areas with limited transportation, resulting in low follow-up compliance Patients and family members have varying educational backgrounds, leading to differing levels of acceptance toward home-based exercise rehabilitation	They have the most fundamental and strongest desire to improve long-term quality of life and cardiac function Widespread use of communication tools like smartphones and WeChat	Develop targeted health education materials using accessible animated explanations of cardiac rehabilitation principles and safety protocols Establish a home rehabilitation resource library featuring video demonstrations, provide real-time guidance and Q&A via WeChat, and distribute activity trackers to monitor exercise safety data in real time Optimize follow-up protocols by integrating assessments and plan revisions during scheduled outpatient visits, reducing patients' trips to the hospital
Context	First implementation of an off-site exercise rehabilitation guidance program, lacking evidence-based implementation experience The department lacks standardized management protocols and operational procedures for home-based exercise rehabilitation following atrial fibrillation radiofrequency ablation. The department lacks dedicated rehabilitation therapists and lacks collaborative workflows and communication mechanisms with the Rehabilitation Department Monitoring, managing, and training multiple wards and medical teams on evidence application increases complexity	Management strongly supports evidence-based practice, with multiple successful precedents within the hospital Hospital administration and relevant clinical departments prioritize this initiative; the Nursing Department has organized specialized training and designated rehabilitation therapists to assist The hospital has established a fully functional public information platform, and patient support networks have been created across all wards	Collaborate with Cardiology and Rehabilitation to develop training manuals for healthcare providers Establish a routine system for joint physician-nurse-technician outpatient clinics and rounds

## 4 Discussion

### 4.1 Evaluation and evidence-based revision of quality indicators for ExCR based on the FAME framework

This study carefully evaluated an antiarrhythmic drug-related quality indicator based on current evidence and clinical practice. The evidence recommends initiating exercise “after two half-lives of the drug in patients taking propafenone”. However, in clinical practice, class III antiarrhythmic drugs such as amiodarone are more commonly prescribed for maintenance therapy following atrial fibrillation ablation. Furthermore, guidelines recommend amiodarone as a first-line agent for maintaining sinus rhythm in atrial fibrillation patients (27), demonstrating superior long-term efficacy compared to propafenone (36). A study demonstrated that the recurrence rate of atrial fibrillation in the amiodarone treatment group after 16 months was 35%, significantly lower than 63% in the propafenone group (*P* < 0.001) (37). Consequently, this evidence was deemed inconsistent with the feasibility and appropriateness principles of the FAME framework and was therefore excluded by the evidence-based practice team.

In the best evidence of exercise rehabilitation, the practical application of ambulatory blood pressure monitoring faces technical limitations. The evidence advises maintaining blood pressure ≤ 200/100 mmHg during exercise in post-ablation patients; however, accurate and continuous measurement during physical activity remains technically difficult with existing devices. In this study, the patients used smartwatches of the same specification to monitor their exercise parameters, but smartwatches were unable to monitor ambulatory blood pressure (38). In addition, sleeveless devices suitable for dynamic monitoring during exercise have insufficient sensitivity to instantaneous changes in blood pressure, making it difficult to meet the actual needs of identifying blood pressure fluctuations induced by exercise (39). Given the inability of current ambulatory blood pressure monitoring technology to ensure both accuracy and patient comfort during exercise, this evidence was deemed incompatible with the Feasibility principle of the FAME framework and was thus excluded by the evidence-based practice team.

It should be noted that the evidence recommends “maintaining heart rate < 110 bpm during exercise in post-ablation atrial fibrillation patients”; however, achieving this target proves challenging in clinical practice. A randomized controlled trial indicated that atrial fibrillation patients exhibit significantly higher heart rates during moderate-intensity exercise compared to healthy individuals (40). Moreover, current exercise heart rate targets are primarily derived from clinical evidence in European and American populations, who generally possess higher baseline physical fitness. Maintaining a heart rate below 110 bpm remains beneficial for this demographic during exercise (35). However, applying this uniform standard to Asian populations, particularly elderly patients, often leads to premature attainment of peak heart rate even under moderate exercise intensity. Therefore, maintaining a strict heart rate limit below 110 bpm may be clinically impractical for many post-ablation atrial fibrillation patients. To balance exercise safety and benefits, the evidence-based team adopted a more lenient heart rate control strategy by raising the threshold from < 110 bpm to < 140 bpm. Studies have shown that maintaining heart rates below 140 bpm effectively sustains sinus rhythm without increasing arrhythmia recurrence risk (41). Moderately relaxing the heart rate limit ensures rehabilitative efficacy while avoiding inadequate exercise intensity due to excessive restriction. Therefore, this evidence was modified and retained.

### 4.2 Analysis of barriers to the evidence-practice gap in exercise rehabilitation after radiofrequency catheter ablation for atrial fibrillation

In clinical practice, healthcare professionals can identify gaps between existing evidence and clinical practice by comparing their activities against review indicators. This process helps pinpoint areas for improvement and facilitates the translation of evidence into clinical application (42). The baseline review findings of this study revealed that among the 20 review indicators assessed, only one achieved a 100% implementation rate. Four indicators had implementation rates below 60%, while the remaining 15 indicators showed zero implementation. These findings indicate that barriers to implementing best evidence practices for home-based exercise rehabilitation after atrial fibrillation radiofrequency ablation encompass three dimensions: at the innovation level, inadequate alignment between external evidence and local clinical practice, coupled with the absence of standardized protocols and supporting assessment tools, represents the primary obstacle to evidence translation (43). At the Recipients' level, healthcare providers primarily face insufficient evidence-based knowledge and skills, high clinical workloads, and concerns about the safety of home-based rehabilitation. Nguyen's research indicates that if individual competencies are not simultaneously enhanced, organizational infrastructure improvements alone are insufficient to support the effective implementation of evidence-based practice (44). This perspective is further corroborated by subsequent research: a convergent parallel mixed-methods study revealed (45) that while healthcare providers generally recognize the value of evidence-based practice, they commonly lack the specialized skills and systemic knowledge required for comprehensive implementation. Furthermore, despite healthcare institutions demonstrating high readiness in hardware and institutional frameworks, frontline staff are burdened with heavy clinical workloads. Ongoing compliance requirements further exacerbate their workload. Since these additional demands lack corresponding material incentives (such as salary increases or additional leave), their motivation and engagement remain insufficient. Concurrently, the patient population is characterized by widespread adherence to traditional notions of “postoperative rest,” insufficient cognition and capacity for active rehabilitation participation, and variations in follow-up compliance due to factors like geography and educational background. The baseline review for this study primarily included elderly patients, a group commonly affected by movement disorders. From a psychological perspective, elderly patients often harbor fears about participating in exercise, worrying that it may trigger adverse events like heart attacks or even death (46). Such cognitive biases significantly inhibit their willingness to engage in exercise rehabilitation. From a physiological perspective, some patients believe “excessive exertion” may cause muscle strains accompanied by pain and fatigue (47), leading them to actively avoid related training. Thus, exercise phobia and its associated negative cognitions are core factors hindering patient participation in exercise rehabilitation. Additional research indicates (48) that exercise fear significantly impacts the intensity and time adherence trajectories of home-based rehabilitation following radiofrequency ablation in atrial fibrillation patients. Particularly within the “slow decline-low adherence group,” exercise fear markedly reduces patients' actual effective exercise duration. Hoffmann et al. (49) further revealed a bidirectional relationship between exercise fear and patients' physical activity levels. At the context level, the effective application of evidence in clinical practice is closely tied to the appropriateness of both the evidence and the organizational environment. As a pioneering off-site rehabilitation program, the lack of implementation experience, standardized management systems, and cross-departmental collaboration mechanisms within the institution collectively hindered the promotion and implementation of evidence-based practice. Leadership plays a crucial role in advancing evidence-based practice, particularly at the departmental level, where the timely provision of necessary resources, training, and interprofessional collaboration is essential.

Despite these challenges, this study also identified key facilitators for implementing home-based exercise rehabilitation. Innovation: The evidence underpinning this change primarily stems from high-level clinical guidelines and expert consensus, ensuring strong scientific validity and authority. Additionally, standardized assessment tools and pre-existing interfaces within hospital information platforms provide technical feasibility for structured integration. Recipients: Healthcare professionals generally possess strong educational backgrounds and implementation foundations, demonstrating high alignment with the value of exercise rehabilitation and a strong subjective willingness to implement. Patients' fundamental desire for improved long-term quality of life, coupled with high adoption rates of modern communication tools, creates favorable conditions for remote guidance and patient education. Context: Hospital leadership's strong support for evidence-based practice, prior successful project precedents, and established information platforms alongside patient community networks collectively form a robust organizational foundation. This ensures interdepartmental collaboration and sustainable routine operations.

### 4.3 Translating exercise barrier analysis into targeted clinical solutions

Action is the core element for successfully implementing evidence-based change, and systematic action strategies provide fundamental assurance for the smooth advancement of transformation. Based on the i-PARIHS framework, this study comprehensively analyzed practice barriers and facilitating factors to develop integrated implementation strategies across multiple levels: innovation, recipients, and context. At the change level, the project conducted localized pilot studies to validate the safety and feasibility of the exercise rehabilitation program within China's clinical context. By embedding evaluation processes into an information system, standardized and normalized operations were achieved. For change recipients, knowledge and behavioral shifts among healthcare providers were facilitated through needs assessments, specialized training, and incentive mechanisms. Research indicates that timely incentives and positive feedback during project participation promote efficient collaboration (50). On the other hand, it developed visual health education materials and constructed a digital home support platform, supplemented by fitness tracker monitoring, to enable dynamic oversight of patient compliance and safety. Research confirms that continuous medical supervision significantly improves long-term patient outcomes (51). At the context level, establishing joint physician-nurse-technician clinics and rounds systems, coupled with co-developed standardized training manuals, provides structural support for sustainable change. Collectively, these strategies form a multi-tiered, dynamic implementation framework designed to systematically advance the deep integration of evidence and clinical practice. However, evidence-based practice is an ongoing process of quality improvement. New barriers may emerge during transformation, necessitating dynamic analysis and timely adjustments to change strategies to enhance recipients' trust in the evidence.

### 4.4 Considerations for implementation feasibility

Implementing this program requires healthcare professionals to spend approximately 0.5 h per week per patient on remote monitoring and follow-up (52). A recent study has shown that digital home-based cardiac rehabilitation for patients with atrial fibrillation following radiofrequency ablation is cost-effective, with an incremental cost-utility ratio of 33,572 RMB per QALY, which is below China's willingness-to-pay threshold (53). Scalability varies depending on the implementation setting: tertiary hospitals can integrate existing resources, whereas community settings may require adjustments to service models. As Cersosimo and colleagues noted, cardiac rehabilitation for atrial fibrillation requires multidisciplinary collaboration among nurses, physical therapists, and other specialists (54).

## 5 Conclusion

This study systematically synthesized the best available evidence on exercise rehabilitation for patients following radiofrequency ablation for atrial fibrillation, encompassing 16 pieces of evidence across four domains: exercise assessment and timing selection, exercise prescription, exercise monitoring, and exercise rehabilitation follow-up. Following evaluation by an expert panel using the FAME framework, 20 review criteria were established, and a baseline review was completed. Analysis based on the i-PARIHS framework revealed multi-level barriers to evidence application, including insufficient evidence localization, inadequate knowledge and skills among healthcare providers, patient misconceptions, and incomplete organizational support systems. Corresponding change strategies were developed to address these barriers, including pilot testing, embedding information systems, multi-modal training incentives, digital patient support, and multidisciplinary collaboration protocols.

However, this study has several limitations. Firstly, its single-center design and relatively small sample size may limit the generalizability of the findings to other healthcare settings. Future multi-center studies with larger samples are needed to validate the results. The modified heart rate threshold (< 140 bpm) was developed for our local population and should not be uncritically generalized to other settings without validation. Secondly, as a project focused on identifying the evidence-practice gap and its barriers, it does not yet provide data on the effectiveness of the proposed action strategies in improving clinical outcomes; this validation is the subject of our planned future research. Furthermore, the measurement of patient compliance relied partly on self-reported data, which is subject to bias. Finally, the developed indicators primarily assess the process of care, and their impact on long-term patient health outcomes requires further investigation. Despite these limitations, this study provides a systematic foundational analysis for implementing and optimizing exercise rehabilitation in this patient population (55–59).

## Data Availability

The original contributions presented in the study are included in the article/Supplementary Material. Data are available from the corresponding authors upon reasonable request.
